# Sedentary Behavior and the Use of Wearable Technology: An Editorial

**DOI:** 10.3390/ijerph17124181

**Published:** 2020-06-12

**Authors:** Nathan O’Keeffe, Jennifer L Scheid, Sarah L West

**Affiliations:** 1 Department of Biology, Trent University, Peterborough, ON K9L 0G2, Canada; nathanokeeffe@trentu.ca; 2 Department of Health Promotion, Daemen College, Amherst, NY 14226, USA; jscheid@daemen.edu; 3 Trent/Fleming School of Nursing, Trent University, Peterborough, ON K9L 0G2, Canada

**Keywords:** chronic disease, wearable technology, self-monitoring, accuracy, physical activity, sedentary

## Abstract

Globally, we continue to face a mounting issue of obesity combined with inactivity; sedentary behaviour is independently associated with poor health outcomes including disease and mortality. As such, exploring ways to try to reduce sedentary behaviour and decrease the risk of diseases is an important area of consideration. The role of wearable technology, such as fitness trackers, to encourage and subsequently increase physical activity is relatively well documented. These devices have been successful at encouraging populations to increase daily activity levels. While time being sedentary is often correlated with physical activity participation, this is not always the case. Therefore, it may be just as important to consider the activity an individual is not doing when evaluating health and well-being. This Editorial will summarize the importance of distinguishing between physical activity and sedentary behaviour. It will also discuss how wearable technology, in the form of fitness trackers, may be used to encourage someone to break up sedentary bouts more often. Finally, we will consider important future research directions.

Consider the physical activity (PA) profile of two individuals: individual A and B. Individual A is a healthy adult who works full-time at a desk job, which requires them to be seated at a computer for most of their day. However, individual A walks to and from work, and makes an effort to take breaks throughout the day to stand up, stretch, and move. Individual A also participates in 150 min of moderate to vigorous physical activity (MVPA) per week by playing in a weekend recreational hockey league and going for daily brisk walks in the evening. Individual B is a healthy adult who plays in the same weekend recreational hockey league as individual A, and also regularly achieves their weekly goal of 150 min of MVPA. Similar to individual A, individual B works at a sedentary desk job. However, individual B commutes to work by car and spends upwards of 7–8 continuous hours per day seated at their desk without any active breaks. After work, individual B spends most evenings on the couch watching television.

At first glance, both individual A and B meet target MVPA guidelines (at least 150 min per week) outlined by many countries and major regulatory bodies, including the Canadian Society for Exercise Physiology [1], the Physical Activity Guidelines for Americans [2], and the World Health Organization [3]. Based on their MVPA participation, both individuals are meeting PA goals associated with improved health outcomes. However, with the addition of lifestyle and sedentary behaviour (SB) information, would individual A and B be considered the same from a health perspective? The answer is likely no; while individual A and B both work at primarily sedentary jobs, they both manage their day differently. Individual A breaks up long periods of SB, whereas individual B does not. Individual B is therefore more sedentary overall, which may result in worse health outcomes.

Historically, someone was considered sedentary if they were not physically active or did not engage in prescribed or planned exercise [4]. SB is defined as any behaviour characterized by an energy expenditure ≤ 1.5 metabolic equivalents (METs), often including sitting or lying down [5]. It is important to distinguish SB from physical inactivity; physical inactivity is defined as an insufficient PA level, which fails to meet the recommended MVPA targets [5]. SB and physical inactivity are often related, such that someone who engages in SB is less likely to participate in PA, and thus may fail to meet MVPA targets. However, as highlighted in the introductory case study example, individuals can satisfy their weekly MVPA goal, yet still lead an overall sedentary lifestyle. This is problematic, because published research highlights that SB (independent of MVPA) is associated with an increased risk of chronic disease, cancers, poor health-related outcomes [4,6,7,8,9,10,11], and mortality [8,12]. Thus, SB may be just as important as achieving (or not achieving) PA targets when we examine health outcomes.

Identifying the independent contribution of SB on health highlights a current limitation of published PA guidelines [1]. While based on evidence that supports increased health and well-being with 150 min of MVPA per week, perhaps a more comprehensive approach to PA guidelines is needed; one that incorporates MVPA suggestions, as well as recommendations related to light PA and breaking up bouts of SB. This universal approach is the basis behind the creation of the Canadian 24-h movement guidelines for children and adolescents [13]. The 24-h movement guidelines not only suggest how much MVPA a child should participate in, but how much sedentary time, light activity, and sleep should be obtained to maximize health [1]. Additionally, lowering levels of motorized transport, reducing extended sitting periods and reducing time spent indoors each day are suggested [14]. While 24-h movement guidelines have been developed specifically for children and adolescents, there are currently no published 24-h movement guidelines for adults. However, we note that data defining acceptable levels of SB are lacking [15], thus creating evidence-based guidelines that incorporate informed SB suggestions is a challenge. Even without SB guidelines, we suggest that individuals should focus on breaking up long bouts of SB as much as possible. In fact, it has been reported that 30 min of SB relocated in the form of light PA results in an improved metabolic profile, including reduced triglycerides and insulin [16]. An even more effective intervention includes the addition of replacing 30 min of SB with MVPA, which results in further improvements to metabolic health [16]. Overall, defining optimal SB amounts and incorporating them into suggestions or guidelines is an area of research that still requires much work.

So is there at tool that might encourage breaking up SB bouts? We suggest that wearable technology may be one technique for measuring and addressing SB. Wearable technology, such as fitness trackers and associated smart phone applications, continue to increase in popularity and accessibility. Wearable technology has topped the American College of Sport Medicine’s (ACSM) annual survey of worldwide fitness trends from 2016 to 2020, with estimations that the wearable technology industry is worth $95 billion USD [17]. However, before further examining the utility of fitness trackers in managing SB, an important distinction is required. There is a notable difference between commercially available consumer grade fitness trackers such as Fitbit vs. research grade accelerometers such as the Actigraph GT3X. Like research grade accelerometers, commercial fitness trackers use algorithms to take the raw data they collect and transform it into understandable activity outcomes. However, these algorithms are not publicly available, and this makes it difficult to directly compare data obtained from research accelerometers [18]. There also may be inaccuracies (such as larger estimation errors) with the commercially available consumer trackers [19], and limitations of wrist worn consumer fitness trackers in measuring activities that involve less wrist motion, such as cycling [20]. However, some research has reported that outcome measures from consumer grade fitness trackers are similar to research grade tools [21].

Even if commercial fitness watches are only moderately accurate in measuring PA, can they still be used as a tool to encourage PA participation for the general population, for non-research purposes? Data indicate that consumer fitness trackers can increase PA participation [22]. However, can fitness trackers also be used to reduce SB? A recent systematic review and meta-analysis reported a reduction in SB of an average of 41 min/day when using computer-based, activity monitors, pedometers, and/or phone prompt interventions [23]. For example, one study used a tri-axial accelerometer system called the Gruve Solution^TM^, which is able to track sedentary time, light-, moderate-, and vigorous-intensity activity during the day, and provide palpitations to the user (feedback), while sending daily data to an online software for analysis. The software then provides accessible visualizations that are easy to understand, providing feedback, goal-setting, and additional self-monitoring tools [24]. The Gruve and its online software includes self-monitoring, goal-setting, and real time feedback to help elicit behavioural change (i.e., less SB) [24]. Another study that used Gruve Soltution^TM^ also saw significantly reduced SB [25]. These data demonstrate that current technology has the potential to directly influence SB. However, the Gruve Solution^TM^ is not as consumer friendly as other commercial fitness trackers, as it requires a subscription and it is not as widely available as monitors such as the Fitbit. Future research that examines the ability of consumer fitness trackers to reduce SB are still needed; however, results using the research grade wearable technologies are promising.

With some evidence to suggest that fitness trackers may be able to help encourage a reduction in SB, we consider how they may be able to do so. Many of today’s fitness trackers include a cluster of behavioural change techniques (BCTs), including self-management strategies, health consequences, goal setting, self-monitoring, and feedback on behaviour, which are known to increase PA and change behaviour in the participating individual [26]. Self-management strategies include a stimulus, such as flashing lights, a vibration, or push notifications which indicate it is time to get up and move. For example, consumer fitness trackers, such as Apple watches have stand goals, prompting users to get up and move for at least 1 min once an hour. Similarly, Fitbit activity monitors/watches also monitor hourly movement goals and will prompt users to move 250 steps once an hour. Many watches also include goal setting coded in the software, so real-time tracking can be done to see improvement. This may include goal setting for steps/day, calories burned by MVPA/day, and stand goals, all of which push the user to perform these tasks every day. This also allows the user to self-monitor their progress, providing feedback on their current behaviour [26]. The compounding nature of BCTs have been shown to increase the magnitude of behaviour change. For example, a meta-analysis in 2016 found that when at least three BCT techniques were used together during a goal setting intervention, significant behavioural changes emerged [27].

Gamification is another way fitness trackers may work to reduce SB. Gamification is defined as applying game mechanics to contexts that are typically not seen as a game [28]. For example, gamification in PA can be viewed as step challenges over a seven-day period where a higher number of steps increases one’s chances of “winning”. Gamification boosts user engagement in PA, where points, badges, and leaderboards have been seen to be highly effective motivators [28]. A recent study showed that users’ step counts increased by 23% during a step competition compared to the baseline [29]. In another study, university level undergraduate and graduate students found that interventions involving gamification apps on mobile phones were an effective way to increase daily step counts (daily step counts increased by 40.2%) [28]. The use of gamification can allow the user to set more significant goals and to be pushed to meet them, while being immersed in social experiences. Studies that examine the impact of gamification on increasing PA are reporting mixed results based on the intervention type or how they measured PA (objective versus self-report), however, gamification is still a promising concept when looking to engage and motivate individuals to become more active [28,29,30]. To our knowledge, there are no published studies that use gamification targeted at directly reducing SB (vs. increasing PA); this is another area of future research.

In conclusion, we know that minimizing SB is important for preventing chronic diseases and reducing mortality risk. This Editorial provides information to suggest that wearable technology, i.e., fitness trackers, may be a tool to help facilitate managing SB. However, while we present data and offer a discussion that largely supports the use of wearable technology to positively influence SB, there is research to indicate that wearable technology may actually have negative motivational consequences in adolescents, and that short-term increases in motivation may be mediated by feelings of guilt, competition, and internal pressure [31]. Therefore, future research needs to consider both the potential positive and negative impact of wearable technology on SB and other aspects of health and wellness. Overall, there are still a limited number of published studies that directly examine the impact of a wearable technology, especially consumer grade fitness trackers, on reducing SB (vs. increasing PA or MVPA). There is a need for randomized control trials that determine the optimal amounts of SB one should incorporate into daily living, examine features available within wearable technology to help users manage SB, and directly examine the impact of a wearable technology on SB (independent of PA/MVPA).
